# Extracellular Release of CD11b by TLR9 Stimulation in Macrophages

**DOI:** 10.1371/journal.pone.0150677

**Published:** 2016-03-08

**Authors:** Dongbum Kim, Te Ha Kim, Guang Wu, Byoung Kwon Park, Ji-Hee Ha, Yong-Sung Kim, Keunwook Lee, Younghee Lee, Hyung-Joo Kwon

**Affiliations:** 1 Center for Medical Science Research, Hallym University College of Medicine, Chuncheon, Republic of Korea; 2 Department of Microbiology, Hallym University College of Medicine, Chuncheon, Republic of Korea; 3 Department of Molecular Science and Technology, Ajou University, Suwon, Republic of Korea; 4 Department of Biomedical Science, College of Natural Science, Hallym University, Chuncheon, Republic of Korea; 5 Department of Biochemistry, College of Natural Sciences, Chungbuk National University, Cheongju, Republic of Korea; College of Medicine, Korea University, REPUBLIC OF KOREA

## Abstract

CpG-DNA upregulates the expression of pro-inflammatory cytokines, chemokines and cell surface markers. Investigators have shown that CD11b (integrin αM) regulates TLR-triggered inflammatory responses in the macrophages and dendritic cells. Therefore, we aimed to identify the effects of CpG-DNA on the expression of CD11b in macrophages. There was no significant change in surface expression of CD11b after CpG-DNA stimulation. However, CD11b was released into culture supernatants after stimulation with phosphorothioate-backbone modified CpG-DNA such as PS-ODN CpG-DNA 1826(S). In contrast, MB-ODN 4531 and non-CpG-DNA control (regardless of backbone type and liposome-encapsulation) failed to induce release of CD11b. Therefore, the context of the CpG-DNA sequence and phosphorothioate backbone modification may regulate the effects of CpG-DNA on CD11b release. Based on inhibitor studies, CD11b release is mediated by p38 MAP kinase activation, but not by the PI3K and NF-κB activation. CD11b release is mediated by lysosomal degradation and by vacuolar acidification in response to CpG-DNA stimulation. The amount of CD11b in the exosome precipitant was significantly increased by CpG-DNA stimulation *in vivo* and *in vitro* depending on TLR9. Our observations perhaps give more insight into understanding of the mechanisms involved in CpG-DNA-induced immunomodulation in the innate immunity.

## Introduction

The integrins are non-covalently-associated heterodimeric transmembrane receptors consisting of α and β chains. The β2-integrins (or CD18-integrins) are known as a specific subfamily of integrins, which is expressed in leukocytes [1]. The members of β2-integrins, which include LFA-1 (CD11a/CD18, αLβ_2_), Mac-1 (CD11b/CD18, αMβ_2_), CR4 (CD11c/CD18, p150.95, αXβ_2_), and CD11d/CD18 (αDβ_2_), share a common CD18 subunit. Most integrins interact with extracellular matrix molecules such as fibrinogen, collagen, and laminin to provide the inflammatory immune responses [2–4]. Integrins mediate outside-in and inside-out signaling involved in several biological processes such as adhesion, cell migration, and growth [5, 6].

CD11b (Integrin αM/ITGAM) is associated with a common β2-integrin subunit CD18 to form a Mac-1/complement receptor 3 (CR3). Mac-1 is highly expressed in a variety of cells such as macrophages, monocytes, dendritic cells, granulocytes and natural killer cells [3, 7–9]. The function of CD11b in immunomodulation seems to be different depending on the cell types [10, 11]. Investigators have shown that CD11b negatively regulates TLR-induced immune responses such as expression of pro-inflammatory cytokines (e.g., TNF-α, IL-6, IFN-β) and major histocompatibility (MHC) class II molecules in the macrophages [10]. Inside-out signals from the phosphoinositide 3-kinase and RapL pathways activated by TLR stimulation induce activation of CD11b. In turn, activation of tyrosine kinases Src and Syk inhibit TLR stimulation cascade. Syk interacts with adaptor molecules MyD88 and TRIF, and induces tyrosine phosphorylation of these proteins, which leads to their degradation by the E3 ubiquitin ligase Cbl-b. In agreement with this response, it was observed that TLR ligands activate NF-κB and interferon pathways more strongly in CD11b-deficient cells than wild type cells. Furthermore, injection of TLR ligands induced more production of IL-6, TNF-α, and INF-β in CD11b-deficient mice than in wild type mice [10]. On the other hand, Ling et al. [11] reported that CD11b positively regulates LPS-triggered signaling responses in myeloid dendritic cells because CD11b enhances TLR4 endocytosis and signaling in the endosomes after LPS stimulation in dendritic cells. This phenomenon was not observed in macrophages. Accordingly, TLR4-triggered responses of dendritic cells were decreased and T-cell activation was impaired in CD11b-deficient mice. Therefore, CD11b negatively or positively regulates immune response in dendritic cells and macrophages, and the differential function may be important to fine-tune the balance between adaptive and innate immune responses induced by TLR ligands.

CpG-DNA, characterized as synthetic oligodeoxynucleotides and bacterial genomic DNA with unmethylated CpG dinucleotides flanked by specific base sequences, has powerful immunomodulatory effects that are capable of activating B lymphocytes, macrophages, dendritic cells [12]. TLR9 recognizes CpG-DNA and the immune system is activated by TLR9-mediated signal pathways after CpG-DNA stimulation [13]. However, the expression and function of CD11b in response to CpG-DNA stimulation has not yet been investigated.

Exosomes are cell-derived 30–100 nm-diameter vesicles found in body fluids as well as in cell culture medium, which contain proteins, miRNAs, functional mRNAs, and lipids [14–16]. Exosomes are directly released from cytoplasmic membranes or derived from multi-vesicular bodies and released by their integration with the cytoplasmic membranes, in various cells including hematopoietic cells and tumor cells [16, 17]. *In vitro* and *in vivo* investigations have shown that exosomes can contribute to intercellular communication [14–16]. The protein components include antigen presenting molecules (MHC class I, MHC class II, and CD1), tetraspanin members (CD9, CD63, and CD 81), adhesion molecules (CD11b and CD54), and costimulatory molecules (CD86) [18]. Therefore, exosomes have been investigated as powerful immunomodulatory agents that are capable of immune activation, immune suppression, and immune surveillance [19, 20].

To elucidate the regulation of CD11b expression by TLR9 stimulation, we examined the effect of CpG-DNA on CD11b expression in mouse macrophage cell line RAW 264.7, and in macrophages originated from mouse peritoneal cavity. Experimental data showed that CD11b is released as a component of exosomes in macrophages by TLR9-mediated signaling pathways, when stimulated with specific phosphorothioate-modified CpG-DNA (PS-ODN).

## Materials and Methods

### Cell culture and Reagents

The mouse macrophage cell line RAW 264.7 was purchased from the American Type Culture Collection (ATCC, Manassas, VA). The cells were maintained in Dulbecco’s modified Eagle’s medium (DMEM) with 10% fetal bovine serum (FBS), 100 U/mL penicillin, and 100 μg/mL streptomycin. Viability of the cells was maintained at greater than 95% by assay using trypan blue dye exclusion. Incomplete Freund’s adjuvants and alum were purchased from Sigma-Aldrich (St. Louis, MO). GolgiStop^™^, a protein transport inhibitor containing monensin, were obtained from BD Biosciences (San Diego, CA). The rabbit anti-CD11b polyclonal antibody was obtained from Novus (Littleton, CO). FITC-conjugated rat anti-mouse CD11b (M1/70) and anti-mouse CD18 (C71/16) antibodies were purchased from BD Biosciences. Rabbit anti-CD81 antibody was obtained from System Biosciences (Mountain View, CA). IKK-2 inhibitor BMS-345541, stress-activated protein kinase (SAPK)/Jun N-terminal kinase (JNK) inhibitor SP600125, MAPK/ERK kinase (MEK) inhibitor PD98059, and p38 inhibitor PD169316 were purchased from Calbiochem (San Diego, CA). Autophagy inhibitor 3-methyladenine; inhibitor of vacuolar type H^+^-ATPase (V-ATPase) bafilomycin A1; protease inhibitors leupeptin, pepstatin A, aprotinin, bestatin, and E-64; metalloproteinase inhibitor TIMP1; PI3 kinase inhibitors wortmanin, LY294002, AS604850, and TGX-221; these compounds were purchased from Cayman Chemical (Ann Arbor, MI). When the inhibitors were used, the cells were pre-incubated with each inhibitor for 1 h, before stimulation with CpG-DNA 1826(S). *Escherischia coli* LPS (Sigma-Aldrich) was dissolved in sterile water and added to the cell culture to obtain the desired concentration. The liposomes cholesterol hemisuccinate (CHEMS) and phosphatidyl-boleoyl-γ-palmitoyl ethanolamine (DOPE) were purchased from Sigma-Aldrich.

### Oligodeoxynucleotides (ODNs)

PO-ODNs (natural phosphodiester CpG-DNA) were synthesized from Samchully Pharm (Seoul, Korea) and PS-ODNs (phosphorothioate-modified CpG-DNA) were synthesized from GenoTech (Daejon, Korea). The CpG-DNA 1826(S) used consists of 20 bases containing two CpG motifs (underlined): TCCATGACGTTCCTGACGTT. The non-CpG DNA 2041 (S) (CTGGTCTTTCTGGTTTTTTTCTGG) served as a negative control. MB-ODN 4531 consisted of 20 bases containing three CpG motifs (underlined): AGCAGCGTTCGTGTCGGCCT [21]. The phosphorothioate version of MB-ODN 4531(O) is MB-ODN 4531(S). The endotoxin of the ODNs was determined by a *Limulus amebocyte* assay (Whittaker Bioproducts, Walkersville, MD, USA). Liposome complexes (Lipoplex) consisting of CpG-DNA encapsulated with phosphatidyl-β-oleoyl-γ-palmitoyl ethanolamine (DOPE): cholesterol hemisuccinate (CHEMS) (1:1 ratio) were prepared as reported previously [22, 23].

### Flow cytometry

Expressions of CD11b and CD18 were analyzed in RAW 264.7 cells by FACScan flow cytometer (BD Biosciences). After stimulation with ODNs and LPS, RAW 264.7 cells were washed with PBS containing 0.1% BSA. To block the Fc receptors, the cells were incubated for 20 min at 4°C with 10 μg/ml of the anti-FcγRII/III antibody (BD Biosciences). The cells were incubated with FITC-conjugated anti-CD11b or anti-CD18 (BD Biosciences) for 1 h at 4°C. We used FITC-conjugated mouse IgG2a κ as an isotype control. The FACS data were analyzed with WinMDI 2.8 FACS software.

### Mice and ODN treatment

Mice were retained under specific-pathogen-free conditions at 20–25°C and in 32–37% humidity. Four-week-old female BALB/c mice were purchased from NaraBiotech Inc. (Seoul, Korea), and BALB/c TLR9 knockout mice were purchased from Oriental Bioservice Inc. (Japan). Our animal studies were performed in accordance with the recommendations in the Guide for the Care and Use of Laboratory Animals of the National Veterinary Research & Quarantine Service of Korea, and all animal procedures were approved by the Institutional Animal Care and Use Committee of Hallym University (Hallym-R2014-10). In our protocol, CO_2_ euthanasia is designated for experimental endpoint when the mice lose >20% of initial body weight or show severe or persistent clinical abnormalities including hunched posture, ruffled hair coat, anorexia, dehydration, pallor, inactivity, difficulty ambulating, tachypnea or dyspnea. In this study, there was no case showing such symptom. The mice were sacrificed under Zoletil 50+Rompun anesthesia, and all efforts were made to minimize suffering. The mice were injected intraperitoneally (i.p.) with either PBS, 50 μg of ODNs, incomplete Freund adjuvants, or alum. The mice were sacrificed 24 h after the injection. The peritoneal cavity fluids and peritoneal macrophages were collected from a mouse body with 10 ml RPMI 1640 culture medium. After the peritoneal cavity fluids were centrifuged at 1500 rpm, supernatants were collected.

### Western blotting

To asses CD11b expression, ODNs (5 μg/ml), Lipoplex (5 μg ODN/ml) or LPS (10 ng/ml) were added to RAW 264.7 cells or macrophage from mouse peritoneal cavity, and incubated at 37°C with 5% CO_2_ for 24 h. The CD11b levels in the cell culture supernatants and cell lysates were then measured using Western blotting. Briefly, the cells were centrifuged at 1500 rpm for 5 min at 4°C and then the supernatants were collected. The precipitated cells were washed with PBS, resuspended in lysis buffer (20 mM Tris-HCl, pH 8.0, 137 mM NaCl, 10% glycerol, 1 mM phenylmethylsulfonyl fluoride, 0.15 U/ml aprotinin, 10 mM EDTA, 10 μg/ml leupeptin, 100 mM NaF, 2 mM Na_3_VO_4_, and 1% NP-40), and incubated for 30 min on ice. The cell lysates were centrifuged at 13,000 rpm for 10 min to remove cell debris, and the supernatants were frozen at −20°C. Protein concentration of the lysates and cell culture supernatants was determined using bicinchoninic acid (BCA) protein assay reagent (Pierce, Rockford, IL). Equal amounts of protein were resolved in 10% SDS-PAGE, and the separated proteins were transferred to polyvinylidene fluoride (PVDF) membranes (Millipore, Bedford, MA, USA). The PVDF membranes were blocked in PBS containing 0.05% Tween 20 and 3% Skim milk for 1 h at room temperature, and incubated with appropriate primary antibody for 2 h. Immuno-reactive proteins were measured by means of a horseradish peroxidase-conjugated secondary antibody (Jackson ImmunoResearch Laboratories, Inc., West Grove, PA) and an enhanced chemiluminescence reagent (Amersham Pharmacia Biotech, Piscataway, NJ).

### Exosome

To analyze the exosomal protein in CpG-DNA-treated cell culture supernatants or CpG-DNA-injected mouse peritoneal cavity fluids, we used the ExoQuick-TC^™^ exosome precipitation solution (System Biosciences). The peritoneal cavity fluids were collected from mice i.p. injected with CpG-DNA (5 μg/mouse). ODNs (5 μg/ml) were added to RAW 264.7 cells or to macrophages from mouse peritoneal cavity and the cell-culture supernatants were collected. The peritoneal cavity fluids and cell culture supernatants were centrifuged at 3000 rpm for 15 min to remove cell debris. The supernatants (10 ml) were mixed with ExoQuick-TC^™^ exosome precipitation solution (2 ml) and stored at 4°C overnight. The mixture was centrifuged at 3000 rpm for 5 min at 4°C to collect the precipitated exosome. RIPA buffer (200 μl) (25 mM Tris-HCl, pH 7.6, 150 mM NaCl, 1% NP-40, 1% sodium deoxycholate, and 0.1% SDS) was added to the exosome pellet and vortexed for 15 s. The exosomal proteins were analyzed by SDS-PAGE and Western blotting using anti-CD11b polyclonal antibody. Anti-CD81 antibody was used to detect CD81 as an exosomal indicator.

## Results

### Effect of CpG-DNA on CD11b expression in macrophage cells

**Fig 1:**
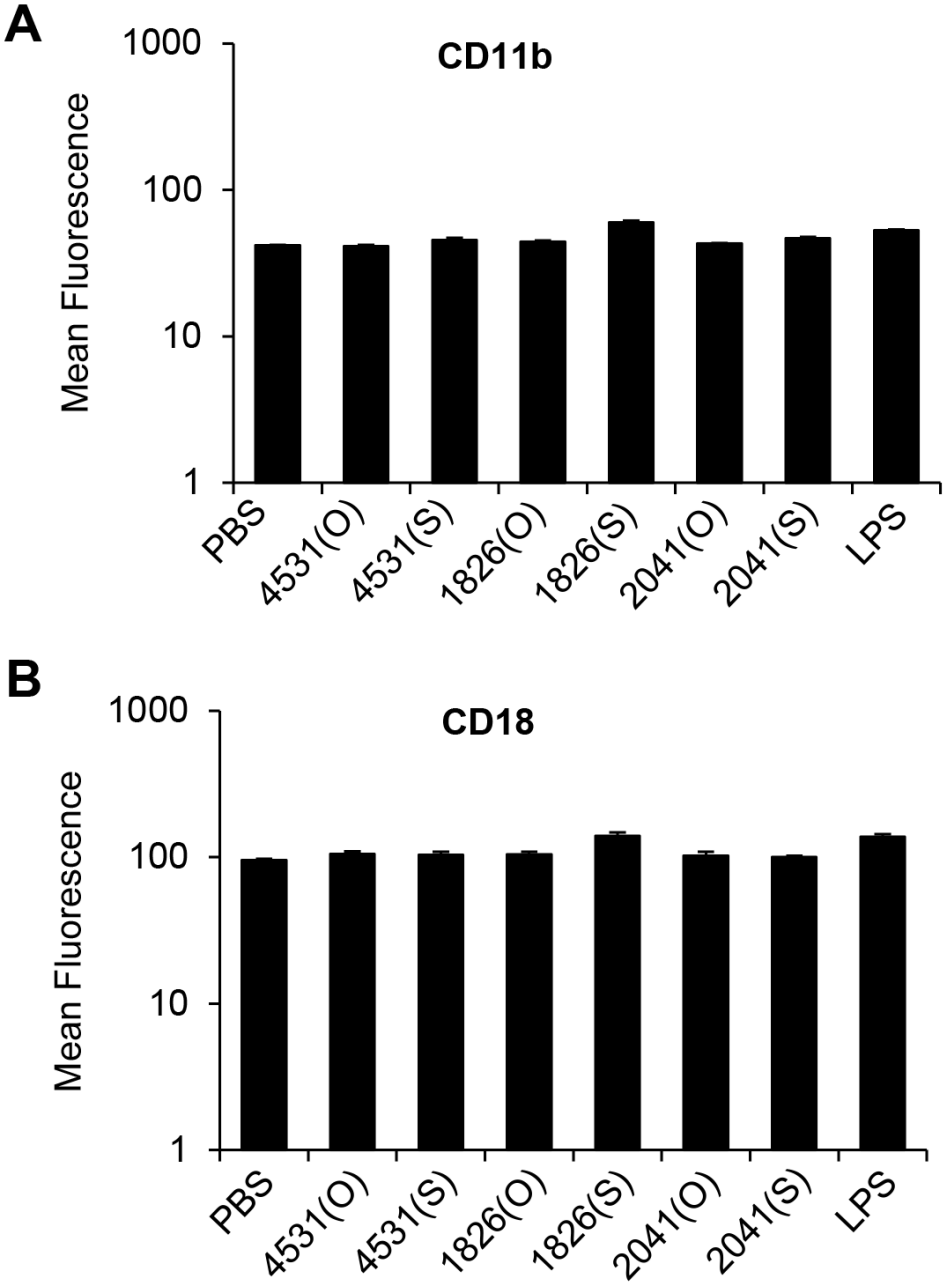
Effect of CpG-DNA on the expression of CD11b and CD18 in RAW 264.7 cells. RAW 264.7 cells were treated with MB-ODN 4531 (4531(O), 4531(S)), CpG-DNA 1826 (1826(O), 1826(S)), non-CpG-DNA 2041 (2041(O), 2041(S)) or LPS for 24 h. After stimulation, RAW 264.7 cells were harvested and the expressions of CD11b (A) and CD18 (B) were analyzed by flow cytometry. Each bar indicates the Mean ± SD of average mean fluorescence intensities (log scale) from three independent experiments. (O), phosphodiester CpG-DNA, (S) phosphorothioate-modified backbone CpG-DNA. For example, the phosphorothioate version of MB-ODN 4531(O) is MB-ODN 4531(S).

Previously, we investigated the effect of CpG-DNA backbone modification (PS-ODN and PO-ODN) on stimulation of innate immunity [24] and we developed immunostimulatory PO-ODN (MB-ODN 4531) from bacterial chromosomal DNA [21]. Here, we compared the ability of CpG-DNAs to induce the expression of CD11b and CD18 using a FACS analysis. We treated RAW 264.7 cells with two different CpG-DNAs (MB-ODN 4531 and CpG-DNA 1826) and a non-CpG-DNA control (non-CpG-ODN 2041) for 24 h. To check the effect of backbone modification, we used both PO-ODN (e.g., MB-ODN 4531(O)) and PS-ODN (e.g., MB-ODN 4531(S)). CD11b and CD18 were expressed on the cell surface of unstimulated RAW 264.7 cells (Fig 1, S1 Fig). The MB-ODN 4531 and non-CpG-DNA control (regardless of backbone type) did not show significant influence on the surface expression of CD11b or CD18. Furthermore, neither CpG-DNA 1826(S) nor LPS induced CD11b and CD18 expression (Fig 1, S1 Fig). Therefore, we can conclude that surface-expression of CD11b is not induced by CpG-DNA or LPS in the macrophage cell line RAW 264.7.

### Release of CD11b in macrophages by CpG-DNA stimulation

**Fig 2:**
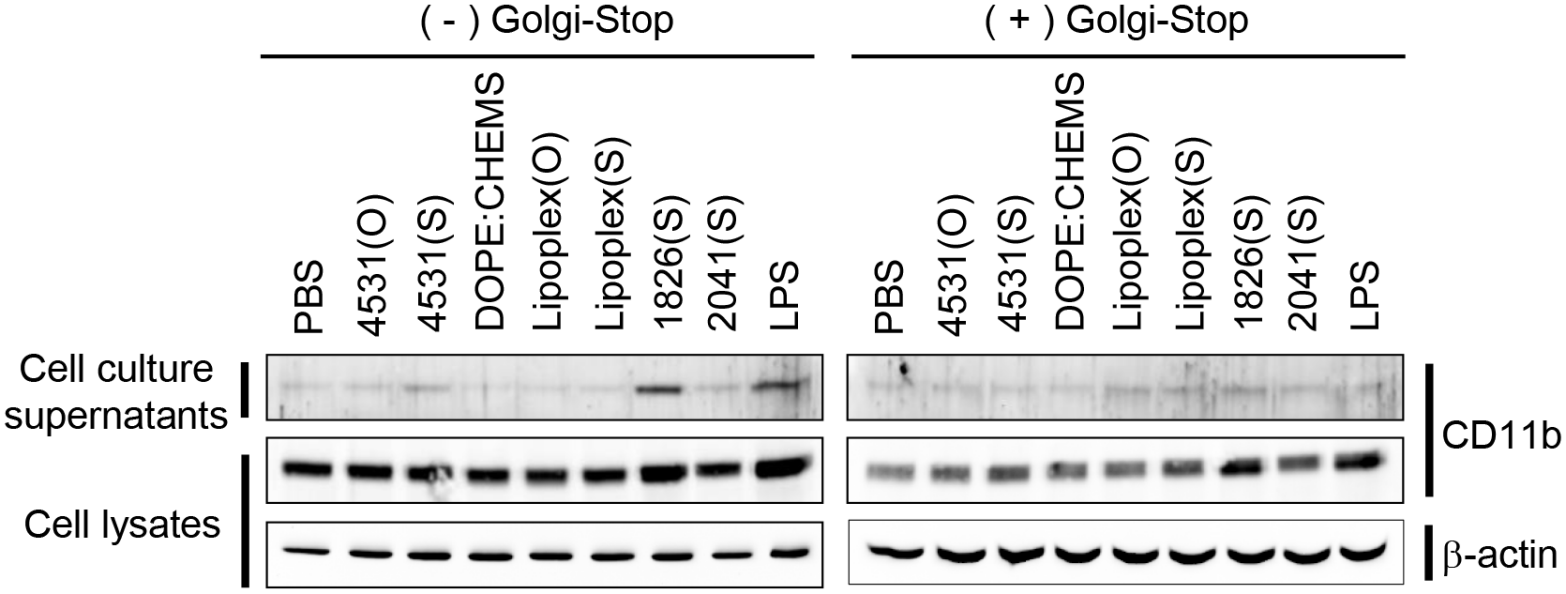
Extracellular release of CD11b in macrophages by CpG-DNA stimulation. RAW 264.7 cells were pretreated with 2 μl GolgiStop^™^, a protein transport inhibitor. The cells were then stimulated with the indicated CpG-DNAs for 24 h. Then, the cell culture media and cell lysates were collected, and the CD11b was monitored by Western blotting. Lipoplex(O), complex of MB-ODN 4531(O) encapsulated with DOPE:CHEMS (1:1 ratio). Lipoplex(S), complex of MB-ODN 4531(S) encapsulated with DOPE:CHEMS (1:1 ratio). β-actin expression level was used as a control. One representative experiment of three is shown.

CD11b was previously found in exosomes from dendritic cells, plasma, and thymus [18, 25, 26]. Therefore, we then investigated whether CD11b is released from macrophages and whether CpG-DNAs can influence the release of CD11b. We treated macrophages with PBS control or various CpG-DNAs, and collected cell lysates and cell culture supernatants for Western blotting analysis. As shown in Fig 2, basal level expression of CD11b was found in the culture supernatant of untreated macrophages, however it was increased after treatment with PS-ODN CpG-DNA 1826(S) or LPS. In contrast, the MB-ODN 4531 and non-CpG-DNA control (regardless of backbone type) failed to induce release of CD11b.

Previously, we examined the effect of CpG-DNAs encapsulated with liposomes on stimulation of innate immunity and confirmed that CpG-DNAs encapsulated in a DOPE:CHEMS complex (Lipoplex) is most effective in humans as well as in mice [22, 23, 27]. Therefore, we treated RAW 264.7 cells with MB-ODN 4531(O) or MB-ODN 4531(S) encapsulated in a DOPE:CHEMS complex (Lipoplex(O) or Lipoplex(S)). However, CD11b was not released after stimulation with Lipoplex(O) or Lipoplex(S) (Fig 2).

To identify if the intracellular transport process is involved in CpG-DNA 1826(S)-induced CD11b release, RAW 264.7 cells were pretreated with GolgiStop^™^, a protein transport inhibitor containing monensin. Then, the cells were treated with various CpG-DNAs or LPS, and the CD11b release was monitored by Western blotting. As shown in Fig 2 (right panel), CD11b release by CpG-DNA 1826(S) and LPS was protected when the cells were pretreated with GolgiStop^™^.

**Fig 3:**
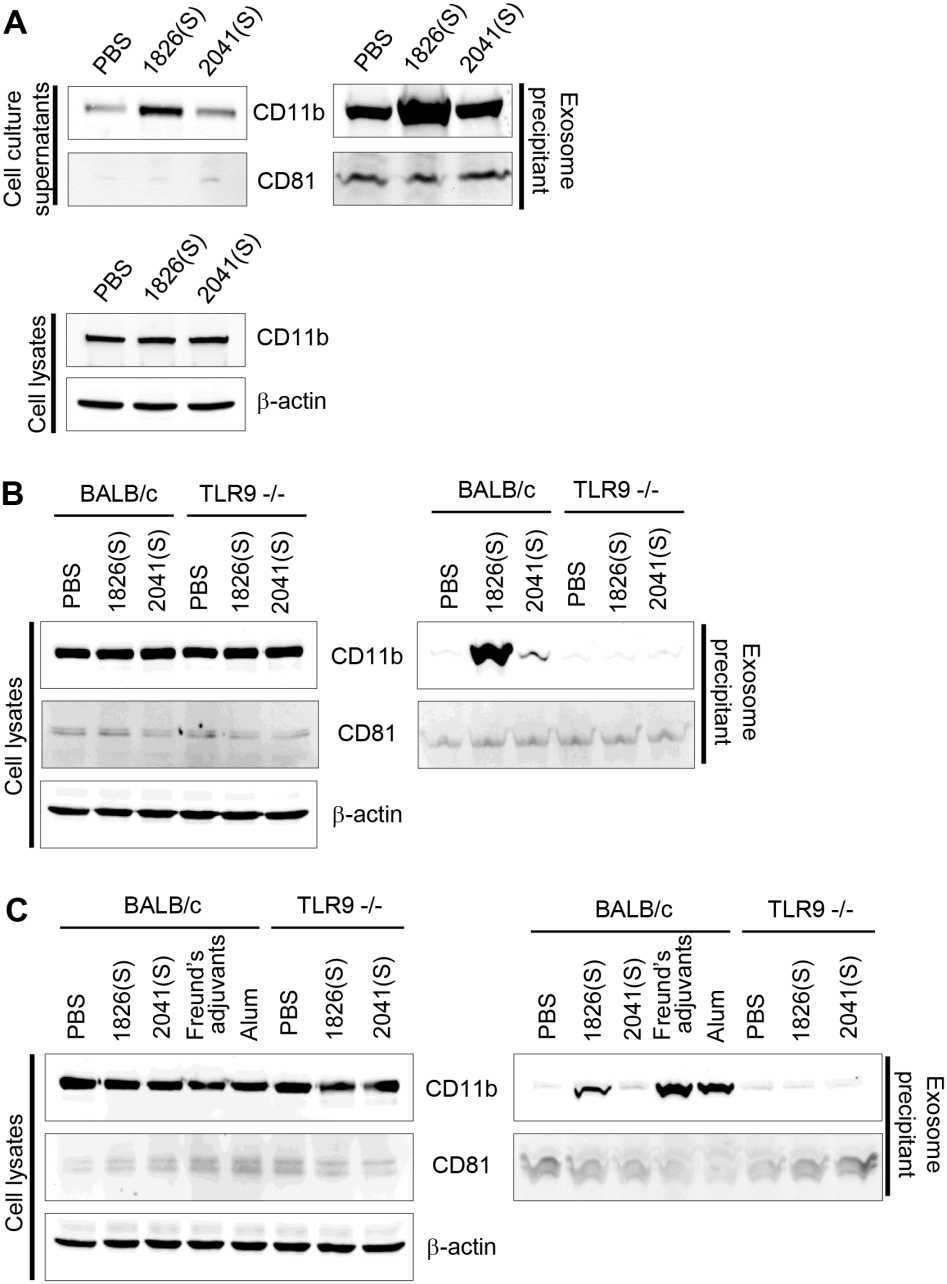
Induction of CD11b release by CpG-DNA stimulation is associated with exosome. (A) RAW 264.7 cells were treated with PBS, CpG-DNA 1826(S) (1826(S)), or non-CpG-DNA 2041(S) (2041(S)) for 24 h. The cell culture media and cell lysates (left panel) were collected, and the CD11b was monitored by Western blotting. β-actin expression level was used as a control. Exosome precipitant (right panel) was collected with ExoQuick-TC^™^ exosome precipitation solution, and the CD11b was monitored by Western blotting. CD81 was used as an exosome control protein. (B) Macrophages obtained from the peritoneal cavity of BALB/c mice and TLR9 knockout mice were treated with PBS, CpG-DNA 1826(S) (1826(S)), or non-CpG-DNA 2041(S) (2041(S)) for 24 h. The cell lysates (left panel) and exosome precipitant (right panel) were collected, and the CD11b was monitored by Western blotting. (C) BALB/c mice and TLR9 knockout mice were i.p. injected with CpG-DNA 1826(S) (1826(S)), non-CpG-DNA 2041(S) (2041(S)), incomplete Freund’s adjuvants, or alum. After 24 h, peritoneal cavity fluids and peritoneal cavity macrophages were collected. CD11b expression in the cell lysates and exosome precipitant was monitored by Western blotting. One representative experiment of three is shown.

To examine directly whether the CpG-DNA-induced CD11b release is mediated via exosome formation, we isolated the exosomes from the CpG-DNA-treated cell culture media and CpG-DNA-injected mouse peritoneal cavity fluids. As shown in Fig 3A, the treatment with CpG-DNA 1826(S) significantly increased the CD11b in the exosome precipitant of RAW 264.7 cell-culture media. Next, the same experiments were performed with macrophages derived from mouse peritoneal cavity. The CD11b was significantly increased in the exosomes of macrophages after CpG-DNA 1826(S) stimulation. There was no significant change in the macrophages derived from TLR9 knockout mice (Fig 3B) demonstrating that CpG-DNA 1826(S) induces release of CD11b in a TLR9-dependent manner.

To confirm further our observation, we decided to evaluate the CD11b release in the peritoneal cavity *in vivo*. We performed i.p. injections into mice with either a PBS control or 50 μg of CpG-DNA 1826(S) or non-CpG-ODN 2041(S), and examined macrophages and the peritoneal cavity after 24 h. As shown in Fig 3C, the injection of CpG-DNA 1826(S) significantly increased the CD11b release in the exosomes of peritoneal cavity, but there was no significant change when injected with non-CpG-ODN 2041(S). The CD11b release after CpG-DNA injection was not observed in TLR knockout mice suggesting the phenomenon is TLR9-dependent. Fig 3C also showed that CD11b release into the peritoneal cavity was observed when the mice were injected with incomplete Freund’s adjuvants and alum.

### Inhibitors of p38 MAP kinase, lysosomal degradation and vacuolar acidification block CpG-DNA induced release of CD11b

**Fig 4:**
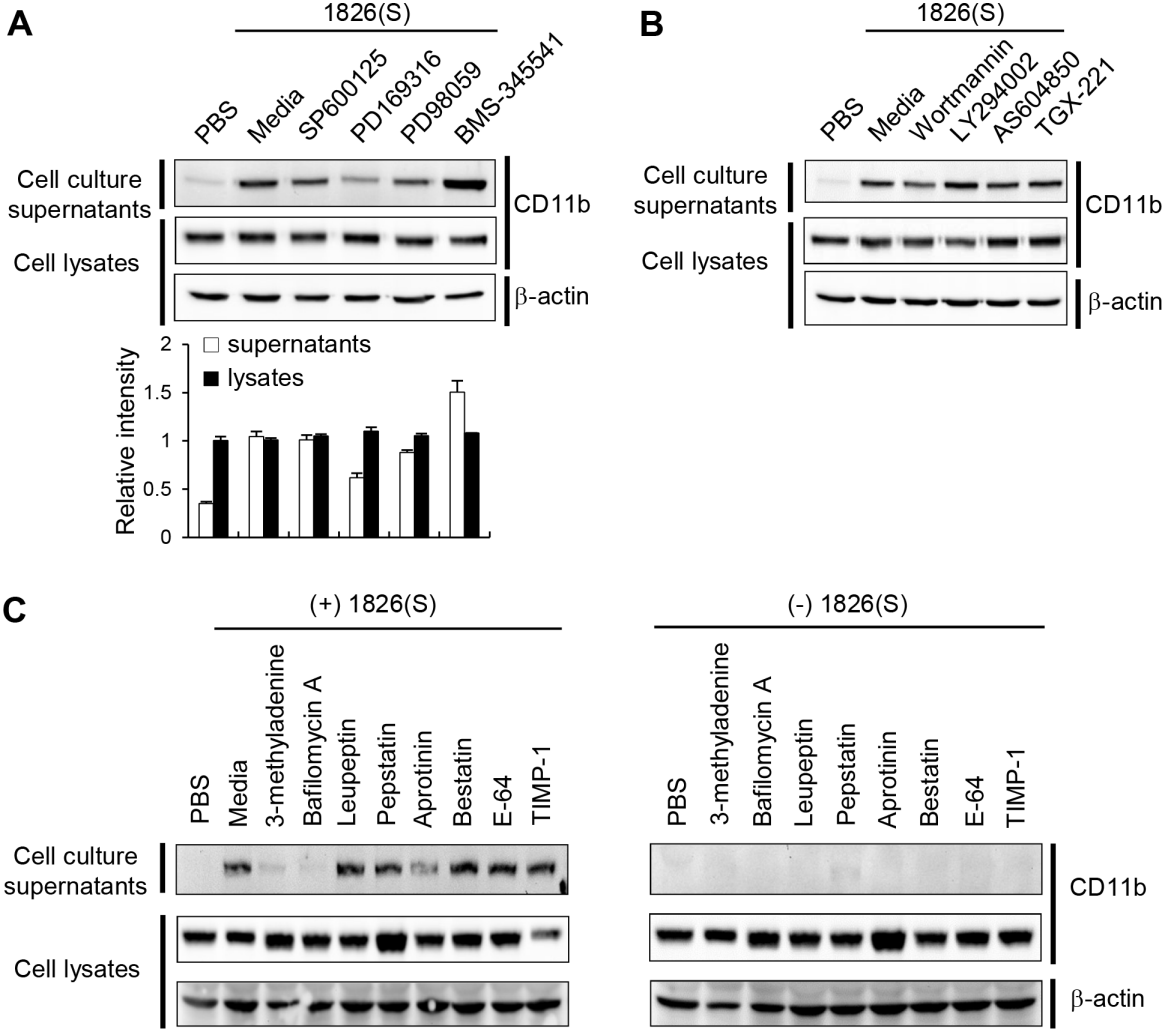
CpG-DNA-induced release of CD11b is linked to p38 MAP kinase activation, lysosomal degradation, and vacuolar acidification. RAW 264.7 cells were pretreated with each inhibitor. The cells were then stimulated for 24 h with CpG-DNA 1826(S) (5 μg/ml). Then, the cell culture media and cell lysates were collected, and the amount of CD11b was monitored by Western blotting. β-actin expression level was used as a control. One representative experiment of three is shown. (A, graph) The band intensity of CD11b in the cell lysates of the media control was taken as 1.0 and relative intensity of the CD11b protein bands in the supernatant and cell lysates compared with the control was calculated. * p < 0.01 (Significant decrease).

Previously, CpG-DNA was reported to activate MAPK superfamily and NF-κB in macrophages [24, 28]. To confirm the contribution of CpG-DNA-mediated MAPK superfamily activation to CD11b release in RAW 264.7 cells, we incubated the cells with or without SAPK/JNK, MEK, or p38 MAP kinase inhibitor for 1 h prior to treatment with CpG-DNA. Exposure of the RAW 264.7 cells to p38 MAP kinase inhibitor (PD169316) significantly blocked the release of CD11b in response to CpG-DNA. Interestingly, the IKK-2 inhibitor (BMS-345541) increased the release of CD11b. In contrast, SAPK/JNK inhibitor (SP600125) and MEK inhibitor (PD98059) had no significant effect (Fig 4A). These results suggest that p38 MAP kinase activation contributes to CD11b release in CpG-DNA-treated RAW 264.7 cells.

Because phosphatidylinositol 3-kinase (PI3K) can be activated by CpG-DNA [29], we next investigated whether pretreatment with PI3K inhibitors block the release of CD11b in CpG-DNA-treated RAW 264.7 cells. However, no significant effect was observed (Fig 4B).

To identify further the signaling pathways involved in CpG-DNA-mediated CD11b release, RAW 264.7 cells were pre-incubated with or without several inhibitors for 1 h prior to treatment with CpG-DNA. As shown in Fig 4C, CpG-DNA-mediated CD11b release was blocked by lysosomal degradation inhibitor (3-methyladenine) and by vacuolar acidification inhibitor (Bafilomycin A1). This further confirms the contribution of endosomal processing in the release of CD11b. Treatment with each inhibitor alone didn’t induce CD11b release.

Taking together these results, we can postulate that the p38 MAP kinase pathway and exosome formation are involved in CpG-DNA-mediated CD11b release in macrophage cell line RAW 264.7.

## Discussion

CpG-DNA induces powerful immunomodulatory responses through the activation of TLR9 [13]. CpG-DNA regulates the expression of cytokines (TNF-α, IL-1, IL-6, IL-12, and IFN-γ), chemokines (MIP-2, MCP-1, RANTES, and IP-10), [12, 30–32], and cell surface markers (CD40, CD80, CD83 and CD86) [12, 33]. Here, we aim to identify the effects of CpG-DNA on the expression of CD11b in macrophages. In contrast to our expectation based on the immune responses induced by CpG-DNA [22, 23, 28, 31–33], we observed no significant induction of CD11b by CpG-DNA stimulation in macrophages (Fig 1).

Generally, the non-bridging oxygens in the backbone of CpG-DNA are substituted with sulfur to make PS-ODN because of its nuclease resistance and efficient uptake into cells. PS-ODN has been studied for application as an immune adjuvant and a therapeutic for infectious diseases [34, 35]. However, adverse effects of PS-ODN have been reported: transient lymphoadenopathy, lymphoid follicle destruction, arthritis, and PS-ODN-specific IgM production [36–39]. To overcome the imperfection of PS-ODN, we screened the natural phosphodiester bond CpG-DNA (PO-ODN, MB-ODN 4531(O)) from *Mycobacterium bovis* genomic DNA, but the immunomodulatory effects of PO-ODN were lower than those of PS-ODN [21]. The activity of PO-ODN is enhanced by liposome-encapsulation: MB-ODN 4531(O) encapsulated in a DOPE:CHEMS (1:1 ratio) complex (Lipoplex(O)) induced effective immune responses such as adjuvant effects and expression of cytokines and cell surface markers [22]. Here, we confirmed that CD11b was released into culture media by CpG-DNAs stimulation in macrophage cell line RAW 264.7 cells. The CD11b was released by PS-ODN CpG-DNA 1826(S) stimulation, but MB-ODN 4531 and non-CpG-DNA control (regardless backbone type) failed to release CD11b (Fig 2A). Further, the release of CD11b was not induced by liposome-encapsulated PO-ODN (Fig 2A). Therefore, differences in the context of the CpG-DNA sequence affect CD11b release, and phosphorothioate backbone modification may regulate the effects of CpG-DNA on CD11b release.

In previous studies, several investigators showed that the exposure of macrophages to CpG-DNA, triggers TLR/IL-1 receptor (IL1-R), resulting in the recruitment of MyD88 and activation of NF-κB and AP-1 for the upregulation of the genes involving innate immune response [24, 28]. CpG-DNA also activated p38 MAP kinase, ERKs and PI3K to induce immune response [29, 40]. In this study, we determined that CpG-DNA-induced CD11b release in macrophages is mediated by p38 MAP kinase activation, but not by PI3K, SAPK/JNK, ERKs and NF-κB activation (Fig 4). We also demonstrated that CD11b release in macrophages is mediated by lysosomal degradation and by vacuolar acidification in response to CpG-DNA stimulation (Fig 4). Most importantly, CD11b release was TLR9-dependent (Fig 3). Further investigation of the precise mechanism in the regulation of CD11b release may lead to the gain of further insight into CpG-DNA function.

Exosomes contain mediators of intercellular communication and immune responses, therefore they have been considered as biomarkers and emerging therapeutics for diseases [19, 20]. In fact, many of the recent studies investigated contribution of exosomes to the immunosuppressive activity of myeloid-derived suppressing cells [18–20]. However, there is limited information regarding biological relevance of exosome in the regulation of TLR-mediated immune responses. Involvement of MicroRNA-29b-containing exosome was implicated in regulating innate and antigen-specific immune response in mouse model of autoimmunity via TLR7 signaling [41]. Exosomes from mycobacterium-infected mice or cultured macrophages contribute to the recruitment and activation of immune cells [42]. Here, we obtained results showing that CD11b is significantly increased in exosome precipitant after stimulation of macrophages with a specific PS-ODN *in vivo* and *in vitro* depending on TLR9 (Fig 3). Expression of CD11b in exosomes was reported previously in dendritic cells [18] and we report CD11b release induced by CpG-DNA for the first time. As CD11b release into the peritoneal cavity was also induced by incomplete Freund’s adjuvants and alum *in vivo*, it is likely that CpG-DNA is one of trigger molecules for the secretion of CD11b in the exosomes of macrophages.

Considering that CD11b negatively regulates innate immunity induced by TLR4 in macrophages [10], the function of CD11b in the regulation of TLR9 signaling has to be determined. One possibility is that release of CD11b via exosomes, induced by CpG-DNA, contributes to excessive TLR9 signaling and side effects of the CpG-DNA in macrophages by excluding CD11b from the cells. To investigate whether this phenomenon is cell type-specific, it is necessary to check expression of CD11b in dendritic cells after stimulation with CpG-DNA. Further examination of these issues has to be done in the future and the information may provide more insight into the mechanisms involved in immunomodulation in innate immunity by CpG-DNA stimulation.

## Supporting Information

S1 Fig Surface expression of CD11b and CD18 is not induced by CpG-DNAs. RAW 264.7 cells were treated with CpG-DNAs or non-CpG-DNAs or LPS for 24 h. Surface expression of CD11b and CD18 was analyzed by fluorescence immunostaining and FACScan flow cytometry. These experiments were performed three times with similar results.
(PPTX)

## Abbreviations

CpG-DNA: synthetic DNA containing immunostimulatory CpG motifs
i.p.: intraperitoneal or intraperitoneally
MB-ODN: CpG-DNA from chromosomal DNA sequences of *M*. *bovis*
ODN: Synthetic oligodeoxynucleotide
PO-ODN: natural phosphodiester CpG-DNA
PS-ODN: phosphorothioate-modified CpG-DNA
