# Supplementary figures and images for: Extracellular Release of CD11b by TLR9 Stimulation in Macrophages
Source: PLoS One. 2016 Mar 8;11(3):e0150677. doi: 10.1371/journal.pone.0150677 (PMC4783063; doi:10.1371/journal.pone.0150677)

## Slide 1
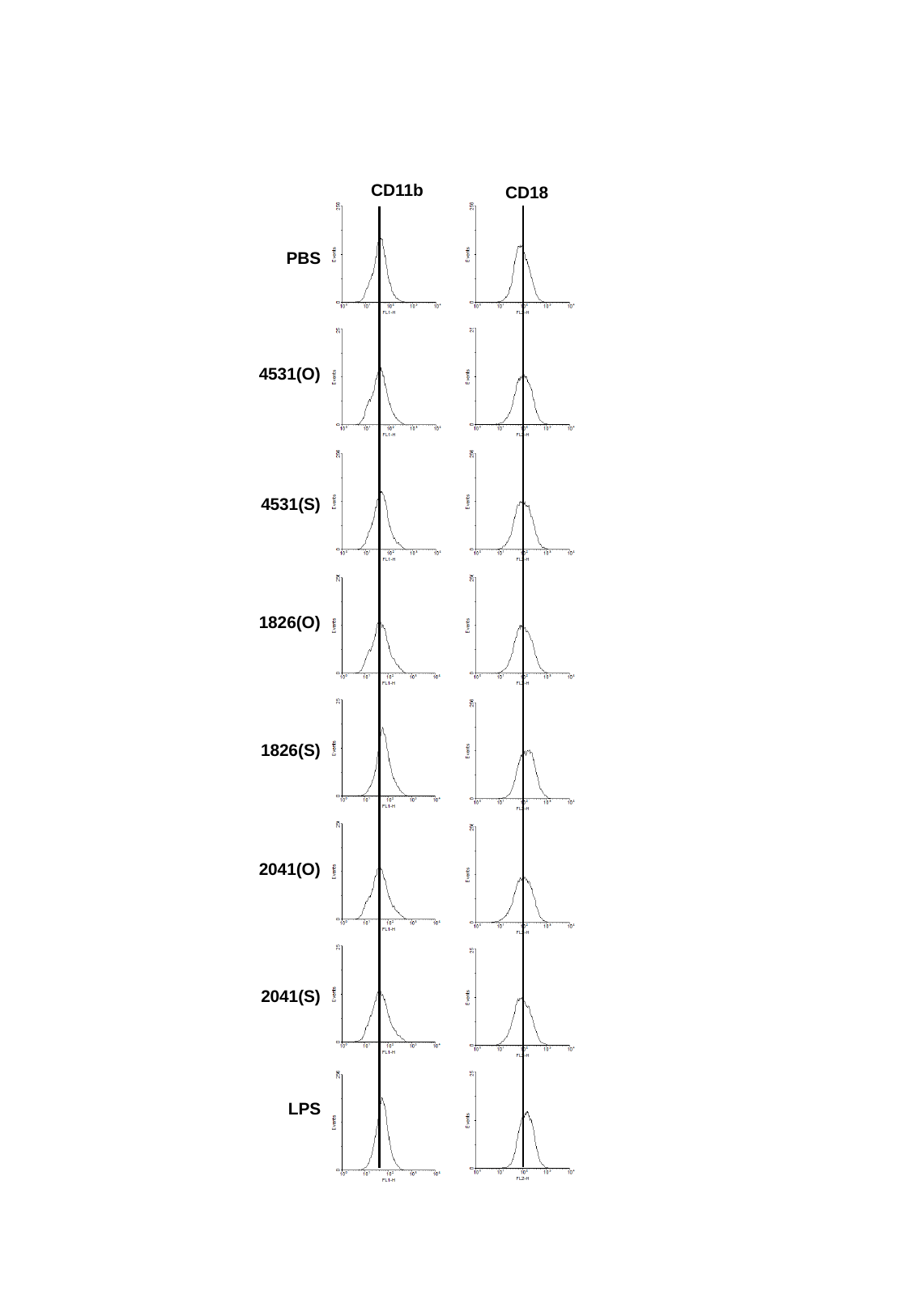

CD11b
CD18
PBS
4531(O)
4531(S)
1826(O)
1826(S)
2041(O)
2041(S)
LPS

Supplement: S1 Fig — RAW 264.7 cells were treated with CpG-DNAs or non-CpG-DNAs or LPS for 24 h. Surface expression of CD11b and CD18 was analyzed by fluorescence immunostaining and FACScan flow cytometry. These experiments were performed three times with similar results. (PPTX) [file pone.0150677.s001.pptx]
